# *Kumkumadi Taila* improves facial skin pigmentation, erythema, and elasticity: an instrument-based exploratory proof-of-concept study with phytochemical profiling for primary skin care

**DOI:** 10.3389/fmed.2026.1796572

**Published:** 2026-04-21

**Authors:** Shyamasundaran Kulangara, Shashank K. S, Deepu Mohanan, Deepthi Viswaroopan, N. S. Reshma, Sushma Naranappa Salethoor

**Affiliations:** 1 Amrita Center for Advanced Research in Ayurveda, Amrita School of Ayurveda, Amrita Vishwa Vidyapeetham, Kollam, Kerala, India; 2 Department of Health Data Science and Artificial Intelligence, McWilliams School of Biomedical Informatics, University of Texas Health Science Center at Houston, Houston, TX, United States; 3 Department of Psychiatry, Kasturba Medical College Mangalore, Manipal Academy of Higher Education, Manipal, Karnataka, India

**Keywords:** ayurvedic dermatology, chemical analysis, claim substantiation, dermalabcombo^®^, facial skin biophysical parameters, *Kumkumadi Taila*, phytochemicalprofiling, skinphysiology/structure

## Abstract

**Introduction:**

*Kumkumadi Taila* is a classical Ayurvedic facial oil traditionally used in primary care as a routine preventive skincare product to enhance the complexion; however, clinical instrument-based data and detailed chemical profiling remain scarce.

**Methods:**

In this single-arm 15-day exploratory study, 30 healthy adults with *Fitzpatrick* skin types III-IV applied *Kumkumadi Taila* once daily to the face using a standardized massage and wash-off procedure, while biophysical skin parameters and safety were monitored at baseline, Day 7, and Day 15 with an integrated DermaLab Combo^®^ device.

**Results:**

Facial melanin index, erythema index, hydration, and elasticity showed statistically significant changes over time, whereas transepidermal water loss and skin thickness did not. This indicates short-term cosmetic improvement without detectable disruption of barrier integrity. Parallel UPLC–MS/MS QTOF analysis of the oil identified multiple bioactive phytochemicals, including safranal, berberine, palmatine, liquiritin, nuciferine, rubiadine, retinol, sesamin, and aliuretic acid, which are consistent with antioxidant, anti-inflammatory, and pigmentation-modulating actions reported in the literature. No adverse events were reported during the study. The results were statistically significant (*p* < 0.05), indicating that the data deviates from a normal distribution and is skewed. Overall, Melanin and Erythema Index decreased significantly, indicating improved pigmentation and reduced redness. Skin elasticity declined over time, while skin hydration fluctuated. TEWL and skin thickness remained stable throughout the study.

**Discussion:**

In this exploratory proof-of-concept assessment, short-term application of *Kumkumadi Taila* was associated with changes in selected facial skin biophysical parameters over 15 days. The study supports its potential as a safe and accessible primary care intervention in the form of a routine preventive skincare product, and its findings highlight the relevance of traditional Ayurvedic topical formulations in routine skin care. The study also demonstrates a practical framework for evaluating non-invasive skin metrics and phytochemicals, serving as a proof of concept.

## Graphical Abstract

**Figure:**
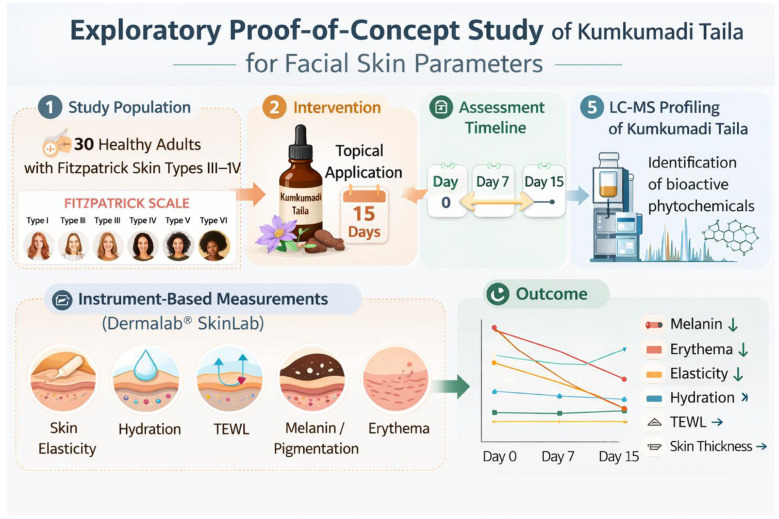

## 1 Introduction

The skin serves as a protective barrier (1, 2) between the human body and the external environment. It safeguards the body from harmful chemical and physical agents, participates in metabolic and thermoregulatory processes, exhibits resorptive functions (3–7), and acts as the first line of defense against pathogenic microorganisms., Additionally, it plays a vital role in immunological responses (8).

Biophysical parameters such as hydration, transepidermal water loss (TEWL), pigmentation, erythema, and elasticity reflect epidermal barrier integrity and overall skin health (2). These measurable indicators are widely used in dermatological and cosmetic research to objectively evaluate skin condition and treatment responses. Alterations in these parameters may arise from aging (9), environmental exposure, or inflammatory processes, thereby influencing skin appearance and function. Once photoaging begins, collagen fibers undergo degradation, leading to skin laxity, wrinkle formation, and pigmentation caused by abnormal melanocyte proliferation. Additionally, elevated levels of matrix metalloproteinases (MMPs) contribute to extracellular matrix degradation, inflammatory cell infiltration, and vascular dilation (10). Prolonged ultraviolet (UV) exposure is regarded as the primary factor responsible for photoaging and the associated structural and functional alterations in the skin (11, 12).

Conventional cosmetic interventions for pigmentation and skin aging include topical depigmenting agents, chemical procedures, and laser-based therapies (13, 14). While effective, some interventions may be associated with adverse effects (15–18), high cost, or limited accessibility. These concerns have contributed to increasing interest in traditional and plant-based skincare formulations for primary care use as cosmetic products, as highlighted in this newspaper article (19).

In Ayurvedic medicine, skin health is considered an indicator of systemic balance and proper tissue nourishment (20, 21). Classical formulations such as *Kumkumadi Taila* have been traditionally used to enhance complexion, reduce pigmentation, and promote skin vitality. “*Kumkumadi Taila*,” named after *Kumkuma* (saffron), which is listed first in the 12 ingredients of this formulation, is one such classical Ayurvedic formulation that has been widely used for ages without reports of any side effects. The daily practice of *Abhyanga* (oil massage) is advocated in Ayurveda for its anti-aging benefits, fatigue reduction, and prevention of *Vata* (one of the three regulatory functional factors of the body)-related diseases, promoting vision clarity, nourishment, longevity, good sleep, and overall skin health (22). Studies suggest that massage can be an effective method for drug delivery by influencing various skin parameters (23). Consequently, the topical application of *Kumkumadi Taila*, followed by a localized massage on the face, is likely to enhance the therapeutic efficacy of the oil through improved drug absorption. *Kumkumadi Taila* is known for restoring normal skin color and complexion. Notably, “*Bhavaprakasha*,” a classical Ayurvedic formulary text, states that visible changes in the skin can occur within seven days of use of this medicated oil (24). This reference serves as the basis for the present study.

The scientific literature review indicates that a significant proportion of the phytochemicals identified in this formulation exhibit dermal absorption (25–28), thereby exerting their therapeutic effects on the skin. However, no studies have evaluated the resulting changes or explored the use of *DermaLab Combo*^®^, a reliable instrument, to apply medicated oil to the skin and objectively assess skin parameters. The *DermaLab Combo*^®^ is a non-invasive, multiprobe skin assessment device widely used in dermatological research. It objectively measures transepidermal water loss (TEWL) using an open-chamber diffusion principle, evaluates skin elasticity through a suction-based deformation method, assesses pigmentation and erythema via optical reflectance analysis, and estimates dermal thickness using high-frequency ultrasound. These instrument-based measurements provide a quantitative and reproducible assessment of skin barrier and structural parameters (29).

A PubMed and Scopus database search using the term “*Kumkumadi Taila”* yielded no results, while a search with “*Kuṅkumādi Taila”* identified one study which examined the physicochemical properties of “*Kumkumadi Ghrita”* and compared the efficacy of “*Nagakesarayukta Kumkumadi Taila”* with “*Kumkumayukta Kumkumadi Taila”* for shelf-life and properties (30). However, the physicochemical properties of *Kumkumadi Taila* have not been studied, nor have any clinical trials been conducted.

This study is a novel attempt to address a crucial gap by objectively measuring changes in facial skin parameters through the application of medicated oil for facial massage, utilizing the *DermaLab Combo*^®^ for precise assessment.

The primary objective of this pilot proof-of-concept study is to explore changes in selected facial skin parameters following topical application of *Kumkumadi Taila* using objective, instrument-based assessment.

The second objective of this study is to analyse the *Kumkumadi Taila* sample used in the clinical trial, aiming to identify the phytochemical constituents with skincare activities.

Phytochemical profiling is conducted using UPLC-MS/MS QTOF to characterize major detectable constituents potentially associated with dermatological activity. Safranal, berberine, palmatine, liquiritin, nuciferine, rubiadine, retinol, sesamin, and aliuretic acid were the constituents detected, which are consistent with antioxidant, anti-inflammatory, and pigmentation-modulating actions, as identified by the literature review.

This exploratory study serves as a proof-of-concept and an exemplar for future research to evaluate the efficacy and effects of Ayurvedic therapies or medicines used for external treatments, incorporating objective and reliable methods. To our knowledge, this is among the first exploratory investigations to integrate objective skin parameter assessment with phytochemical profiling of *Kumkumadi Taila*.

## 2 Material and methods

*Kumkumadi Taila* was procured from a reputed market-available brand that complies with Good Manufacturing Practices (GMP) and meets the laboratory testing requirements prescribed by the Drug Control Cell of the Ministry of AYUSH. All the trial medicine procured was from a single batch of manufacture.

### 2.1 Phytochemical profiling of *Kumkumadi Taila*

Ultra-performance liquid chromatography coupled with tandem mass spectrometry and quadrupole time-of-flight (UPLC-MS/MS QTOF) analysis is a highly sensitive and accurate technique employed in this study to identify and quantify compounds in the *Kumkumadi Taila* formulation. This method integrates the high-resolution separation capabilities of UPLC with the advanced mass spectrometric detection provided by MS/MS and QTOF, ensuring precise analysis of complex mixtures.

#### 2.1.1 Reagents and chemicals

LCMS-grade acetonitrile and methanol were purchased from *Supelco (Germany)* and *Merck (Germany)*, respectively. Formic acid, 99%, was obtained from *Merck (Germany)*. All chemicals used were of analytical grade unless stated otherwise. Ultrapure, deionised water was produced in-house with the Milli-Q system (Millipore; resistance ≥ 18.5 MΩ)

#### 2.1.2 Sample preparation

A 2-mL aliquot of *Kumkumadi Taila* was transferred to a 2-mL microcentrifuge tube, and an equal volume of a 1:1 methanol solution was added. The mixture was thermomixed at 1,000 rpm for 1 h at 60 C. The aqueous layer was then carefully transferred into a 25-mL volumetric flask and filled to volume with a 1:1 methanol solution. From this, 1 mL was taken and reconstituted in a separate 10-mL volumetric flask, made up to volume with a 1:1 methanol solution to obtain a sufficient dilution. The solution was then filtered through a 0.2-μm syringe filter and transferred to a screw-cap vial. The characterization was performed using a 6545 UPLC-MS/MS QTOF system (Agilent, USA).

#### 2.1.3 UPLC MS/MS QTOF conditions

An LC-MS/MS system consisting of a 6545 QTOF UPLC-MS/MS QTOF *(Agilent, USA)* was used for the analysis. *Agilent MassHunter Qualitative Analysis 10.0* software was used for data processing and interpretation. The analytical column was a *Zorbax Eclipse* C18 column (3.0 × 100 mm; 2.7 μm), operated at 37 °C. The mobile phase consisted of solvent A (1% v/v formic acid in water) and solvent B (acetonitrile). A linear gradient program was performed, starting with 2% B for 0–2 min, increasing to 20% B at 3 min and 30% B at 14 min; at 24 min the gradient reached 50% B, and 98% B was maintained for 1 min, then reduced to 75% B and 25% B at 34 and 38 min, respectively. Finally, the gradient was returned to 2% B for 1 min, giving a total run time of 40 min. The flow rate was set to 0.25 mL/min. The electrospray-ionization (ESI) source operated in positive mode, with sheath gas set to 60 psi and a capillary voltage of +2,000V. Nitrogen was used as the collision gas, and the source temperature was 325 C. Data acquisition and compound identification were carried out in multiple reaction monitoring (MRM) mode.

### 2.2 Exploratory proof-of-concept clinical study

#### 2.2.1 Approvals

The exploratory study protocol received approval from the Institutional Ethics Committee (IEC), and the study was registered with the Institutional Ethics Committee (IEC), reference number IEC No. IEC.ASA.UGR.03, and the Clinical Trial Registry of India (CTRI), reference number CTRI/2023/07/054637. Before enrollment, all participants provided voluntary written informed consent.

#### 2.2.2 Recruitment

##### 2.2.2.1 Selection of participants

The participants enrolled in the study were healthy volunteers, as the primary objective was not to treat established pigmentary disorders or age-related loss of elasticity but to objectively evaluate if the trial drug could bring about any change concerning Varnya (enhancement of skin complexion) as described in Ayurvedic literature. Kumkumadi Taila is traditionally indicated for improving skin tone and complexion; therefore, healthy young adults were considered appropriate for assessing its physiological effects on normal skin. The predominance of participants in the 20–25-year age group allowed evaluation of subtle early physiological changes while minimizing confounding factors such as intrinsic aging, chronic dermatological conditions, or hormonal variations that are more common in older populations.

##### 2.2.2.2 Selection of sample size

This study was designed as a single-arm, open-label, exploratory proof-of-concept clinical study including 30 participants. As the study aimed to generate preliminary evidence to inform the design of future trials, a formal sample-size calculation was not conducted. The sample size of 30 (31) was selected in line with commonly recommended ranges for pilot studies (*n* = 20–40), which are considered sufficient for estimating variability and preliminary effect sizes. A control arm was therefore not included, as the study was intended to provide initial evidence to guide the design and sample size estimation of subsequent adequately powered randomized controlled trials.

##### 2.2.2.3 Study setting

Thirty healthy participants, students and staff volunteers from the *Amrita Vishwa Vidyapeetham campus, Amritapuri*, where the clinical trial was conducted, were recruited for the study through local advertisements. Eligibility screening was conducted at the School of Ayurveda by a designated physician.

#### 2.2.3 Inclusion and exclusion criteria

The study included healthy individuals of either gender aged 18–45 years willing to participate and to abide by the protocol. Participants in the study were those with *Fitzpatrick* skin types III or IV and who were non-smokers. They consented to avoid direct sunlight and to refrain from using cosmetics, sunscreens, and undergoing any cosmetic therapies during the trial period. They also committed to avoiding a diet rich in carotenoid-rich foods throughout the study. The exclusion criteria for this study encompassed participants with active skin infections on the face, individuals with known allergies to cosmetics, moisturizing agents, or other skin treatment products, and those currently using over-the-counter medications for skin conditions. Additionally, participants who were consuming intoxicants, participating in other clinical trials, or objecting to comply with the protocol were also excluded. Also, individuals with any condition that might impair their ability to provide voluntary, fully informed consent were excluded from the study.

#### 2.2.4 Dropouts

There were no dropouts in the clinical trial participants.

#### 2.2.5 Trial medicine

*Kumkumadi Taila* was procured from a reputed market-available brand that complies with Good Manufacturing Practices (GMP) and meets the laboratory testing requirements prescribed by the Drug Control Cell of the Ministry of AYUSH. All the trial medicine procured was from a single batch of manufacture. The preparation and indications of *Kumkumadi Taila* are described in texts such as *Ashtanga Hridayam* (32), *Bhaishajya Ratnavali* (33), and *Chakradatta* (34). Although minor variations exist in the preparation methods and ingredients described across different classical texts, the pharmaceutical company from which the medicine was obtained has adhered to the formulation and procedure prescribed in the *Ashtanga Hridayam*. This formulation comprises twelve ingredients, viz., Kumkuma (Crocus sativus), Chandana *(Santalum album)*, Rakta Chandana *(Pterocarpus santalinus)*, Padmaka *(Nelumbo nucifera)*, Padmakesara *(Nelumbo nucifera)*, Utpala *(Nymphaea caerulea)*, Nyagrodha *(Ficus benghalensis)*, Plaksha *(Ficus virens)*, Yastimadhu *(Glycyrrhiza glabra Linn.)*, Usheera *(Chrysopogon zizanioides)*, Goat milk *(Capra aegagrus hircus)*, and Sesame oil *(Sesamum indicum)*.

#### 2.2.6 Procedures

Screened subjects were enrolled in the study and instructed to apply 3–5 drops of *Kumkumadi Taila* on their faces after washing with water and patting dry. They were instructed to gently massage the oil into their skin for 5 min and leave it on for an additional 15 min. They were instructed to rinse it off with green gram powder *(Vigna radiata)* as a cleansing agent following oil application. This powder is traditionally used in Ayurveda to remove excess oil; it primarily acts through gentle mechanical cleansing and mild saponin-mediated surfactant activity. While the plant contains various phytochemicals, their biological effects are mainly reported in concentrated extracts or systemic use. In this study, topical application as a briefly applied cleansing paste (50–60 s) is unlikely to permit meaningful percutaneous absorption. Therefore, its contribution to the observed changes in skin parameters is expected to be negligible (35, 36).

Participants were required to follow this procedure once daily in the morning for 15 days. Long-term structural changes in dermal collagen architecture and stable melanin remodeling generally require several weeks to months. However, classical Ayurvedic literature describes this formulation as producing visible improvement in skin complexion within a short duration. In particular, the *Bhavaprakasha* (24) states that perceptible changes in skin appearance may occur within seven days of regular use, and this traditional reference served as the conceptual basis for selecting the study timeline. Accordingly, outcome assessments were conducted at Day 0, Day 7 (midpoint based on the classical claim), and Day 15. Although traditional texts suggest visible effects within 7 days, we extended the observation period to 15 days to evaluate whether early changes persisted or progressed over time.

#### 2.2.7 Safety assessments

Subjects were monitored for adverse events (AEs) throughout the study. Drug safety and tolerability were evaluated based on detailed records of AEs, with a focus on the types and frequencies of both common and serious AEs. A serious AE was defined as an AE that was fatal or life-threatening or required prolonged inpatient hospitalization. However, no AEs were reported by any of the volunteers during the study period.

### 2.3 Statistical analysis

The obtained data were coded, tabulated, and analyzed using *SPSS Version 20* for *Windows*. Descriptive statistics were expressed as Mean ± Standard Deviation (SD) for continuous variables. Normality of the data was assessed using the *Shapiro-Wilk* test and visual inspection of histograms and Q-Q plots. Comparison of skin parameters (melanin, erythema, hydration, TEWL, ultrasound skin thickness, and elasticity) across time points (Baseline, Day 7, and Day 15) was performed using Repeated Measures ANOVA for normally distributed data and the *Friedman test* for non-normally distributed data. Pairwise comparisons were conducted using the paired *t*-test or the *Wilcoxon* Signed-Rank test as appropriate. A *p*-value of less than 0.05 was considered statistically significant.

## 3 Results

### 3.1 Results of UPLC MS/MS QTOF analysis

**Figure 1:**
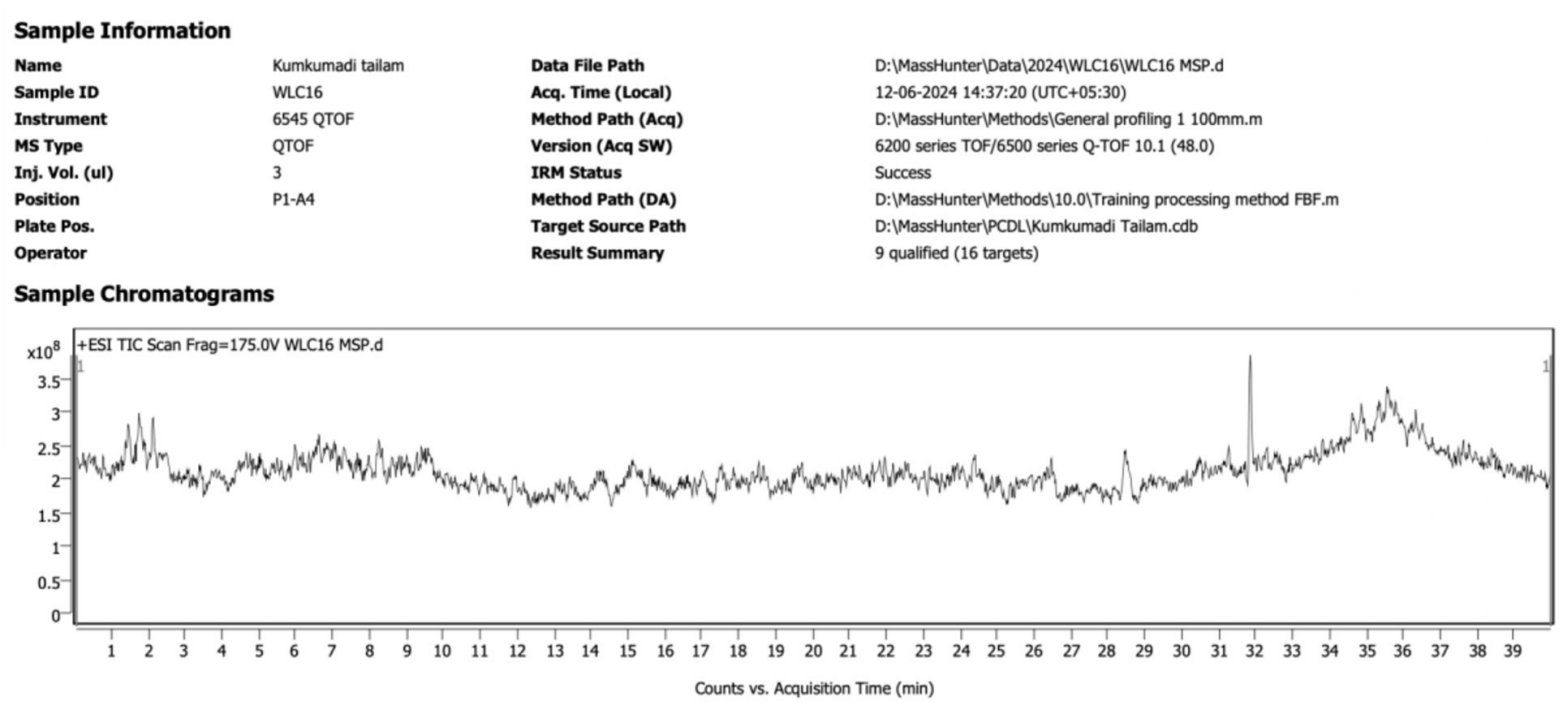
Total ionic chromatogram of *Kumkumadi Taila* obtained using UPLC MS/MS QTOF. UPLC MS/MS QTOF, Ultra Performance Liquid Chromatography-Tandem Mass Spectrometry with Quadrupole Time-of-Flight.

Our work aimed to identify the phytochemicals having skin care activity present in *Kumkumadi Taila*, and we have endeavored to identify the specific phytochemicals derived from herbs during the preparation of *Kumkumadi Taila*. A detailed literature survey provided 16 phytochemicals that show skincare activities. Using the above 16 phytochemicals, we had created a UPLC MS/MS QTOF Library using PCDL software. *Kumkumadi Taila* was further subjected to UPLC MS/MS QTOF analysis. Figure 1 shows the total ion chromatogram (TIC) obtained using UPLC-MS/MS QTOF. We employed the find-by-formula (FBF) technique to determine the chemical components present in the sample. Quadrupole Time-of-Flight (QTOF) investigation identified a total of six compounds from *Kumkumadi Taila*. The findings of this analysis are provided in Table 1.

**Table 1:** The Phytochemicals identified from *Kumkumadi Taila* using UPLC-MS/MS QTOF.

Sl. No	Compounds	Molecular formula	RT (Retention time)	M/Z
1	Safranal	C_10_H_14_O	29.388	151.1114
2	Berberine	C_20_H_18_NO_4_	32.097	337.1243
3	Palmatine	C_21_H_22_NO_4_	32.113	353.1586
4	Aliuretic acid	C_16_H_32_O_5_	22.942	305.2332
5	Liquiritin	C_21_H_22_O_9_	37.812	419.1346
6	Nuciferine	C_19_H_21_NO_2_	32.678	296.1653
7	Rubiadine	C_15_H_10_O_4_	34.871	255.0652
8	Retinol	C_20_H_30_O	35.752	287.2369
9	Sesamin	C_20_H_18_O_6_	34.638	355.1135

### 3.2 Results of clinical study

#### 3.2.1 Demographic data

The demographic and baseline characteristics of the study population reveal that males constitute the majority, accounting for 60% (18 individuals), while females make up 40% (12 individuals). Most participants (83%, 25 individuals) belong to the 20–24-year age group, with the remaining 16% (5 individuals) aged between 25 and 26 years. In terms of BMI distribution, 63.33% (19 individuals) fall within the healthy weight range, 20% (6 individuals) are overweight, 13.33% (4 individuals) are underweight, and only one participant (3.33%) is categorized as mildly obese. Regarding dietary habits, a larger portion of the group (73.33%, 22 individuals) follows a non-vegetarian diet, while 26.66% (8 individuals) adhere to a vegetarian diet. In the assessment of *Fitzpatrick* skin type, 63.33% (19 individuals) are classified as Type 3, typically representing lighter skin tones that tan after sun exposure, and 36.66% (11 individuals) as Type 4, indicative of darker skin tones that tan easily and rarely burn. Overall, the study population was predominantly male, young (mainly 20–24 years old), and of healthy weight, with most participants following a non-vegetarian diet and having Fitzpatrick skin type III. The results are depicted in Table 2.

**Table 2:** Baseline demographic and clinical characteristics of participants (*N* = 30).

Characteristic	Category	*n*	%
Gender	Male	18	60
	Female	12	40
Age (years)	20–24	25	83
	25–26	5	17
BMI category	Underweight	4	13
	Healthy weight	19	63
	Overweight	6	20
	Class I obesity	1	3
Food habit	Vegetarian	8	27
	Non-vegetarian	22	73
*Fitzpatrick* skin type	Type III	19	63
	Type IV	11	37

#### 3.2.2 Descriptive statistics of skin parameters at different time points

The *Kolmogorov-Smirnov* and *Shapiro-Wilk* tests for normality were conducted for all skin parameters at Baseline, Day 7, and Day 15. The results were statistically significant (*p* < 0.05), indicating that the data deviates from a normal distribution and is skewed. Therefore, non-parametric tests are appropriate for further analysis. Skin elasticity measurements at all three time points were constant and therefore omitted from the normality test (refer Table 3)

**Table 3:** Descriptive statistics of skin parameters at different time points.

Parameter	Baseline (Median, IQR)	Day 7 (Median, IQR)	Day 15 (Median, IQR)
Melanin index	37.35 (43.6–31.2)	34.50 (38.2–28.5)	34.50 (36–30.63)
Erythema index	13.65 (14.2–10.4)	13.30 (13.8–13.3)	11.90(13.8–11.9)
Skin hydration	222 (414.25–190)	218 (283.5–194.75)	220 (555–213)
TEWL	25.1 (25.3–14.08)	13.35 (35.2–12.3)	34.5 (34.5–12.1)
Skin thickness	885 (1,433–655)	1,034 (1,526–930.5)	1,125 (1,435–610)
Skin elasticity	0.2 (0.2–0.1)	0.1 (0.1–0.1)	0.1 (0.2–0.1)

IQR, Inter Quartile Range. Median (IQR) is reported due to non-normal data distribution.

A *Friedman* test was performed to examine the differences in skin parameters over three time points: Baseline, Day 7, and Day 15. The results indicated a statistically significant change in Melanin Index (χ^2^ = 49.186, *p* = 0.000), Erythema Index (χ^2^ = 29.309, *p* = 0.000), Skin Hydration (χ^2^ = 15.724, *p* = 0.000), and Skin Elasticity (χ^2^ = 13.975, *p* = 0.001). However, TEWL (χ^2^ = 2.690, *p* = 0.261) and Skin Thickness (χ^2^ = 1.800, *p* = 0.407) did not show significant differences across time points (refer Table 4).

**Table 4:** *Friedman* test for differences in skin parameters over time.

Parameter	Chi-Square (χ^2^)	df	*p*-value	Interpretation
Melanin index	49.186	2	0.000^*^	Significant
Erythema index	29.309	2	0.000^*^	Significant
Skin hydration	15.724	2	0.000^*^	Significant
TEWL	2.690	2	0.261	Not significant
Skin thickness	1.800	2	0.407	Not significant
Skin elasticity	13.975	2	0.001	Significant

*Friedman* test, *p* value < 0.05^*^.

*Post-hoc Wilcoxon* Signed-Rank tests with *Bonferroni* correction were conducted to identify specific time-point differences. The Melanin Index significantly decreased from Baseline to Day 7 (*Z* = 4.791, *p* = 0.000), continued to decrease significantly from Baseline to Day 15 (*Z* = 2.958, *p* = 0.003), and showed further reduction from Day 7 to Day 15 (*Z* = 4.356, *p* = 0.000). Similarly, the Erythema Index significantly decreased at each time point comparison, indicating continuous improvement in skin redness.

Skin Hydration significantly decreased from Baseline to Day 7 (*Z* = 2.937, *p* = 0.003), but no significant differences were observed between Baseline and Day 15 or between Day 7 and Day 15, suggesting fluctuating Hydration levels. TEWL and Skin Thickness did not exhibit significant differences across time points, indicating stability in these parameters. However, Skin Elasticity significantly decreased from Baseline to Day 7 (*Z* = 3.047, *p* = 0.002) and from Day 7 to Day 15 (*Z* = 2.858, *p* = 0.004), but the difference between Baseline and Day 15 was not significant (refer Table 5).

**Table 5:** *Post-hoc Wilcoxon* signed-rank test for pairwise comparisons.

Parameter	Baseline vs. Day 7 (*Z* score, *p* value)	Baseline vs. Day 15 (*Z* score, *p* value)	Day 7 vs. Day 15 (*Z* score, *p* value)
Melanin index	4.791, 0.000^*^	2.958, 0.003^*^	4.356, 0.000^*^
Erythema index	2.725, 0.006^*^	2.377, 0.017^*^	3.179, 0.001^*^
Skin hydration	2.937, 0.003^*^	1.209, 0.190	0.318, 0.750
TEWL	2.236, 0.025^*^	1.641, 0.101	1.845, 0.065
Skin thickness	0.567,0.571	1.887, 0.059	0.72, 0.943
Skin elasticity	3.047, 0.002^*^	0.905, 0.366	2.858, 0.004^*^

*Wilcoxon* signed ranks test, *p* value < 0.05.

**Figure 2:**
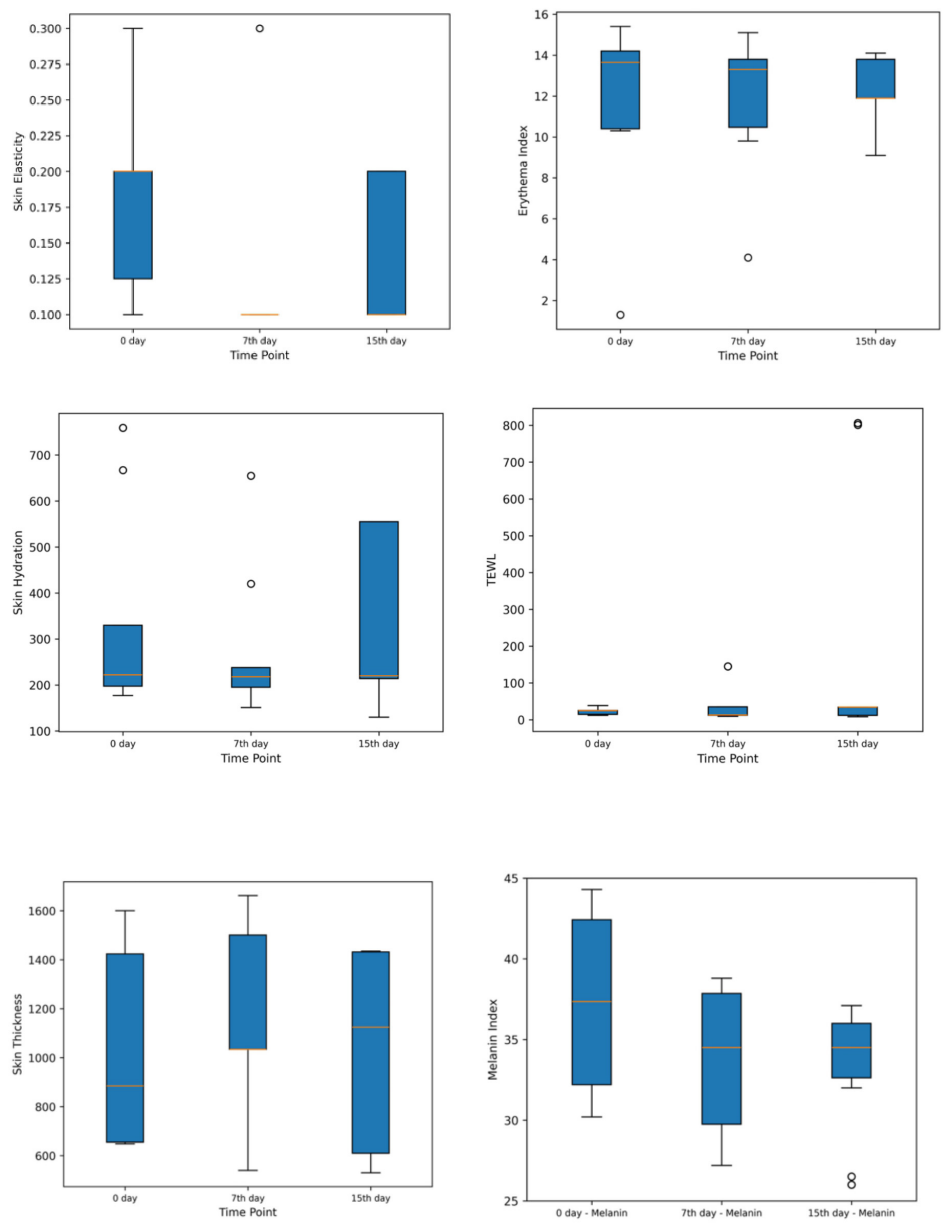
Box plots showing skin color, skin color erythema, hydration, TEWL, ultrasound skin thickness and elasticity. TEWL, Trans-Epidermal Water Loss.

Overall, the results suggest that Melanin and Erythema Index decreased significantly, reflecting improvements in pigmentation and skin redness. Skin Elasticity also declined over time, while Skin Hydration showed some fluctuation. TEWL and Skin Thickness remained stable throughout the study (Refer to Figure 2).

## 4 Discussion

This exploratory clinical and phytochemical profiling evaluated the preliminary associations observed in the use of *Kumkumadi Taila* in improving facial skin parameters using an objective, instrument-based approach using *DermaLab Combo*^®^ and profiled its phytochemical composition using UPLC-MS/MS QTOF. The findings provide quantitative evidence supporting classical Ayurvedic claims while offering modern mechanistic explanations for the observed benefits.

### 4.1 Phytochemical profile and mechanistic insight

A literature review of the ingredients of *Kumkumadi Taila* formulation indicates a wide range of bioactive phytoconstituents and related compound classes, including flavonoids, polyphenols, lignans, terpenoids, fatty acids, and vitamins, have been reported to exert beneficial effects on skin physiology.

The literature review on the phytochemicals from the ingredients which have action specific to skin conveyed that *Kumkuma (Crocus sativus)* contains crocin, which decreases oxidative stress (37), suggesting potential as an anti-aging agent. *Chandana (Santalum album)* possesses santalol compounds that show significant tyrosinase-inhibitory activity for complexion enhancement, while α-santalol exhibits strong radical-scavenging properties (38, 39). *Rakta Chandana (Pterocarpus santalinus)* contains Santalin, which contributes to anti-inflammatory effects primarily through ROS scavenging, helping in skin repair (40). *Padmaka (Nelumbo nucifera)* (41, 42) and *Padmakesara (Nelumbo nucifera Gaertn)* (43, 44) include flavonoids known to suppress tyrosinase and collagenase enzymes, thereby supporting skin tone and structure. *Utpala (Nymphaea caerulea)* (45, 46)*, Nyagrodha (Ficus benghalensis)*, (47) and *Plaksha (Ficus virens)* (48) are rich in flavonoids and procyanidins that exert antioxidant and anti-inflammatory effects and inhibit melanogenesis. Goat milk *(Capra aegagrus hircus)* exhibits anti-allergic, anti-inflammatory, and antioxidant activities, providing a soothing base for the formulation (49, 50). Sesame oil *(Sesamum indicum)* possesses sesamin, which protects the skin from UV radiation (51) and sesamolin, and sesaminol act as antioxidants (52). *Yastimadhu (Glycyrrhiza glabra Linn.) shows an anti-inflammatory, antibac-terial and antioxidant activities* (26). Vetiverol, β-Vetivone, α-Vetivone, Khusimol, Epizizanol phytochemcials of *Usheera (Chrysopogon zizanioides)* show anti-inflammatory and antioxidant activity (53) (refer Table 6).

**Table 6:** Ingredients of *Kumkumadi Taila* skin care-related phytoconstituents identified from literature review.

Sl no.	Sanskrit name	Botanical name	Active phytochemical	Pathway	Activity	Ref
1	*Kumkuma*	*Crocus sativus*	Crocin	Potential against ROS, NF-κB inhibition pathway, MAPK and STAT1 modulation, glycan-mediated signaling regulation.	Anti-oxidant & Anti-inflammatory	(37)
			safranal		Anti oxidative, Anti agening	(54)
				Inhibiting hyaluronidase, elastase, and collagenase, enzymes responsible for degrading the skin	Photoaging	(55) (56)
				UV-blocking potential		(25) (57)
2	*Chandana*	*Santalum album*	Santalol	Act as a tyrosinase inhibitor	Promotion of a fair complexion	(38)
			α-Santalol	The oil inhibits the oxidative enzyme 5-lipoxygenase and has DPPH radical scavenging activity	Anti-oxidant	(39)
			β-Santalol	Inhibits 5-LOX, COX, IL-17, PDE4, tyrosinase, Disrupts microbial membranes, reduces cytokine release	Anti-inflammatory	
3	*Rakta Chandana*	*Pterocarpus santalinus*	Santalin	ROS-scavenging and NF-κB–dependent inflammatory-gene suppression, with possible contribution from MAPK pathway modulation.	Anti-inflammatory activity	(40)
			Santalin B			
4	*Padmaka*	*Nelumbo nucifera*	hyperoside	Suppression of tyrosinase protein	Anti-melanogenesis activity	(41) (42)
			astragalin			
			nuciferine	Suppression of TNF-α-mediated NF-κB signaling via inhibition of p65 phosphorylation and demonstrating tyrosinase inhibitory activity that may contribute to its therapeutic potential in hyperpigmentation disorders.		(64) (65)
5	*Utpala*	*Nymphaea caerulea Savigny*	myricetin 3-O-(3″-O-acetyl)-α-l-rhamnoside	Acts by limiting ROS-induced inflammation	Antioxidants and Antiaging activity	(45) (46)
			myricetin 3-O-α-l-rhamnoside			
			myricetin 3-O-β-d-glucoside			
			quercetin 3-O-(3″-O-acetyl)-α-l-rhamnoside			
			quercetin 3-O-α-l-rhamnoside			
			quercetin 3-O-β-d-glucoside			
			kaempferol 3-O-(3″-O-acetyl)-α-l-rhamnoside			
			kaempferol 3-O-β-d-glucoside			
			naringenin			
			(S)-naringenin 5-O-β-d-glucoside			
			isosalipurposide			
			β-sitosterol			
			β-sitosterol palmitate			
			24-methylenecholesterol palmitate			
			4α-methyl-5α-ergosta-7,24(28)-diene-3β,4β-diol			
			Ethyl gallate			
			Gallic acid			
			p-coumaric acid			
			4-methoxybenzoic acid			
6	*Nyagrodha*	*Ficus benghalensis Linn*.	Kaempferol	Free radical scavenging; inhibition of protein denaturation.	Antioxidant, and Anti-inflammatory activity	(47)
			Quercetin	Radical scavenging via hydroxyl groups.		
			Apigenin	p53 signaling pathway.		
			Quercetin-3-galactoside	DPPH scavenging activity		
			Rutin	Not mentioned		
7	*Plaksha*	*Ficus virens*	B-type Procyanidins (PC), Prodelphinidins, Propelargonidins (PP), Afzelechin / epi-afzelechin, Catechin, Epicatechin	enzyme inhibition, substrate competition, metal-chelation, and ROS-mediated suppression of melanogenesis. They chelate the dicopper ions in the enzyme's active site and reduce o-quinones (enzyme products) back to colorless compounds. (pathway not specifically mentioned for each phytochemical)	Tyrosinase inhibitors	(48)
8	*Padmakesara*	*Nelumbo nucifera Gaertn*	myricetin-3-O- glucoside (Myr-3-Glc), rutin, quercetin-3-O- glucuronide (Quer-3-Glu), kaempferol-3-O- robinobioside (Kae-3-Rob), kaempferol-3-O- glucoside (Kae-3-Glc), kaempferol-3-O- glucuronide (Kae-3-Glu), isorhamnetin-3-O- glucoside (Iso-3-Glc)	Binds to the catalytic Zn^2+^ site of collagenase, disrupting its metal-ion binding and inhibiting activity. They occupy the tyrosine-substrate binding pocket and interferes with the CuA/CuB histidine-coordinated catalytic center of tyrosinase.	inhibits collagenase and tyrosinase enzymes	(43) (44)
9	Goat milk	*Capra aegagrus hircus*	presence of medium-chain fatty acids, conjugated linoleic acid, short-chain fatty acids, vitamins A, C and E	Pathway not mentioned	Anti-allergic	(49) (50)
					Anti-inflammatory,	
					Antioxidant	
					Antimicrobial properties	
10	*Sesame oil*	*Sesamum indicum*	Sesamin	Inhibiting the MAPK/NF-kB pathways.	Anti-inflammatory effects	(51) (66) (51) (70) (71)
			sesamolin, and sesaminol		Antioxidant	(52)
11	*Yastimadhu*	*Glycyrrhiza glabra Linn*.	Liquiritin	Upregulation of antioxidant enzymes and modulation of reactive oxygen species (ROS)–mediated inflammatory pathways, thereby potentially limiting UV-B–induced photodamage	Antioxidant	(26) (61) (62) (63) (73)
12	*Usheera*	*Chrysopogon zizanioides*	Vetiverol, β-Vetivone, α-Vetivone, Khusimol, Epizizanol		Anti-inflammatory, Antioxidant	(53)

However, LC–MS analysis in the present study detected nine major phytoconstituents: safranal, liquiritin, sesamin, nuciferine, rubiadin, berberine, palmatine, retinol, and aliuretic acid. These phytoconstituents are known to contribute significantly to the formulation's therapeutic activity for skin hydration, reducing pigmentation, and enhancing overall skin health.

Safranal can reduce oxidative stress and replenish superoxide dismutase, suggesting its potential as an anti-aging agent (54). The literature indicates that safranal may diminish photoaging by inhibiting hyaluronidase, elastase, and collagenase, enzymes responsible for degrading the skin (55, 56). While some studies suggest UV-blocking potential (25, 57), these properties require further validation in topical oil formulations.

Similarly, Berberine has been reported to suppress matrix metalloproteinases-9 and−1 in keratinocytes following ultraviolet radiation, supporting its utility in combating skin aging (58, 59). Additionally, Palmatine can quench singlet oxygen, demonstrating strong antioxidant properties (60).

Regarding pigmentation and inflammation, liquiritin has been reported to reduce oxidative stress through upregulation of antioxidant enzymes and modulation of reactive oxygen species (ROS)–mediated inflammatory pathways, thereby potentially limiting UV-B–induced photodamage (61–63). Nuciferine demonstrates tyrosinase inhibitory activity and has shown therapeutic potential in hyperpigmentation, with molecular docking studies suggesting effective binding to tyrosinase enzymes involved in melanin synthesis (64, 65). Rubiadin (66) and sesamin (51) also exhibit antioxidant and anti-inflammatory properties that may help reduce collagen degradation and photodamage associated with ultraviolet exposure.

**Figure 3:**
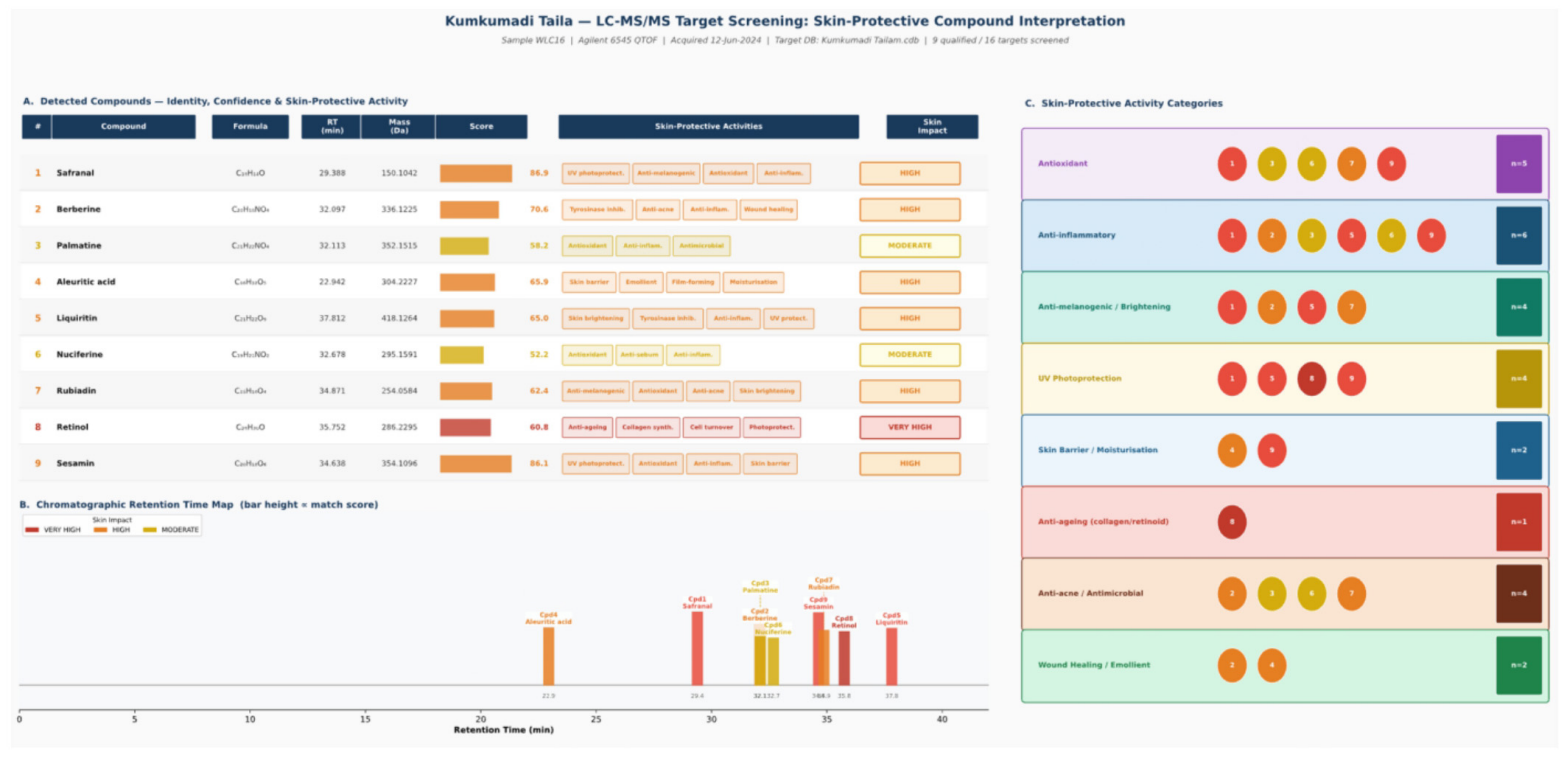
LC-MS target screening: skin protective compound interpretation.

Collectively, the presence of these bioactive phytoconstituents in the formulation may contribute to its observed effects by acting through complementary mechanisms, including reduction of oxidative stress, modulation of inflammatory pathways, inhibition of melanin synthesis, and protection of dermal structural proteins. Such combined actions may help improve measurable biophysical skin parameters such as pigmentation, erythema, and elasticity (refer Figure 3).

### 4.2 Detection caveat

While crocin *(*in *Crocus sativus)*, santalol *(*in *Santalum album)*, and santalin *(*in *Pterocarpus santalinus)* are repeatedly reported in the literature and mechanistically relevant to the observed clinical outcomes, they were not directly identified in our untargeted UPLC–MS/MS QTOF readout. This non-detection most likely reflects (i) sub-quantifiable abundance under our extraction and acquisition settings (below LOD/LOQ), and/or (ii) absence of these molecules in the available MS/MS spectral library, which prevents confident library matches in untargeted mode. Definitive confirmation typically requires authenticated marker standards (for retention-time and fragmentation matching) and/or targeted acquisitions (e.g., PRM/MRM/SRM) or standard-addition spiking. Accordingly, the mechanistic roles we attribute to crocin (37), santalol (38), and santalin (40) are supported by prior reports but remain analytically inferential in the present dataset pending targeted confirmation.

### 4.3 Clinical effects on skin parameters

Statistically significant improvements were observed in the melanin index, erythema index, and skin elasticity over the 15-day application period.

#### 4.3.1 Melanin Index

Median melanin index decreased by ~7.6% within the first week and ~9.8% by day 15, indicating visible brightening of skin tone. Such depigmenting effects align with the *Varnya* property (complexion enhancer) described in classical Ayurvedic texts such as *Ashtanga Hridayam* (67), *Bhaishajya Ratnavali* (68), and *Chakradatta* (34), and are supported by modern evidence that safranal, identified in the oil, reduces oxidative stress and inhibits collagenase/elastase, protecting against photoaging (25, 54).

Safranal identified through LCMS MS/MS QTOF analysis, reduces oxidative stress and inhibits enzymes such as elastase and collagenase, which are critical in the aging process (54). It also offers protection against photoaging by boosting antioxidant activity (69) and enhancing sun protection when encapsulated in liposomes or solid lipid nanoparticles (25, 56). Sesamin mitigates the effects of prolonged exposure to ultraviolet (UV) radiation from sunlight, which triggers the production of reactive oxygen species (ROS) and accelerates skin photoaging (51, 52). This clinical trial demonstrated a significant reduction in melanin levels containing phytochemicals such as Safranal, Liquiritin, Sesamin, Nuciferine, and Rubiadin. Liquiritin promotes cell survival, decreases oxidative stress and inflammation, and protects skin from UV-B damage by upregulating antioxidant enzymes and collagen synthesis while lowering ROS and cytokine secretion (61–63). Sesamin, from sesame oil, shields the skin from UV radiation and reduces inflammation (70), especially due to the presence of lignans (71), and protects collagen by inhibiting the MAPK/NF-kB pathways.

#### 4.3.2 Erythema index

Redness reduced by ~12.8% over the study period, indicating anti-inflammatory and soothing activity, which helps in prolonging the cell senescence (72). Liquiritin (73), sesamin (70), and berberine, also present in *Kumkumadi Taila*, are known to downregulate inflammatory mediators and support skin barrier repair (69, 74).

#### 4.3.3 Skin elasticity

A time-dependent reduction in skin elasticity was observed, with significant decreases during the early and mid-study periods, although elasticity values at Day 15 did not differ significantly from baseline. As skin elasticity is a key indicator of youthful skin, these findings suggest transient changes over time (75). Elasticity was assessed using the suction-based *DermaLab Combo*^®^ (76), in which lower deformation amplitude under suction corresponds to increased resistance to distension and therefore improves firmness. (77). In the context of 15 days of topical *Kumkumadi Taila* application, the early reduction in distensibility may reflect short-term modulation of stratum corneum hydration, lipid layer reinforcement, and surface tightening effects due to repeated oil application and massage. The return of elasticity values toward baseline by Day 15 suggests physiological adaptation of the skin rather than sustained stiffening or impairment. Thus, the findings may indicate transient enhancement in skin firmness during the intervention period, without adverse alteration of intrinsic elastic properties.

#### 4.3.4 Skin hydration

An initial ~23.3% rise in hydration was seen by day 7, although this plateaued thereafter. Such transient gains may result from the occlusive and emollient nature of the oil medium, which reduces water loss and temporarily increases skin suppleness (78). While hydration improved initially (Day 0 to Day 7), it plateaued thereafter, suggesting that while *Kumkumadi Taila* enhances hydration, it may require a longer duration or adjunct measures for sustained improvement. Another potential contributing factor is the demographic characteristics of our study population, specifically the university students, who often experience prolonged academic schedules, potentially leading to inadequate hydration due to decreased water intake (79).

#### 4.3.5 TEWL and skin thickness

No significant change was observed, suggesting *Kumkumadi Taila* maintains barrier integrity without causing excessive water loss or dermal disruption, an important safety attribute for topical products (75).

### 4.4 Adverse events (AEs)

No adverse events related to the application of *Kumkumadi Taila* were reported during the study period, supporting the formulation's tolerability. The adverse effects (AEs) of the identified compounds demonstrate varying profiles. Safranal, aliuretic acid, nuciferine, and rubiadine have no reported side effects or adverse events in the current literature. More than ≥ 4 g of berberine may lead to dermatological reactions, such as erythema, irritation, or allergic responses, in a subset of individuals (74). Topical application of sesame has demonstrated protective effects against UV radiation and has also been shown to possess chemopreventive properties (68, 70). The study showed that UVA irradiation (4 J/cm^2^) of HaCaT keratinocytes in the presence of palmatine (50 μM) resulted in a 50% reduction in cell viability, indicating that palmatine may increase photosensitivity and thereby raise the risk of phototoxic reactions (80). Retinol is known to induce a “retinoid reaction,” characterized by pruritus, burning, erythema, and desquamation, particularly during initial use or at elevated concentrations of 2% (81). It also enhances susceptibility to ultraviolet-induced damage, necessitating photoprotection during use (82).

### 4.5 Probable mode of action of *Kumkumadi Taila* with Ayurvedic rationale

The key ingredients of *Kumkumadi Taila* include saffron, *Khas* grass, red sandalwood, Indian madder, and liquorice. These components are well-known for their *Pitta*-pacifying properties and for enhancing the quality of blood (83–85), which is crucial for addressing skin discolouration.

The ingredients used in this formulation, such as *Kumkuma*- Crocus sativus, according to Ayurveda, possess properties such as *Rakta Prasadana* (enhancement of blood quality) (85). *Ushira - Chrysopogon zizanioides*, exhibits *Kushtahara* (anti-dermatosis) properties, along with being *Varnya* (complexion enhancer) and having *Sheeta Virya* (cold potency) (83). *Yashtimadhu - Glycyrrhiza glabra Linn.*, is known for its *Varnya* and *Snigdha* (unctuous) properties, along with having *Sheeta Virya* (84). The combination of these ingredients contributes to improving skin quality parameters, as evidenced by the outcomes of this study. A formulation that helps in pacifying *Pitta* and *Vata* also improves the overall quality and texture of the skin. From a clinical point of view, the individual ingredients and the synergetic action of this formulation reaffirm this action.

### 4.6 Implications and significance

This appears to be the first study to assess *Kumkumadi Taila* using a combination of classical application protocol, instrument-based skin measurement, and advanced phytochemical fingerprinting. The formulation produced measurable cosmetic benefits—~10% improvement in skin tone, ~13% reduction in redness, and improved firmness—within 15 days, without compromising skin barrier function. These results validate traditional usage while demonstrating potential for integration into evidence-based dermatology and the development of natural cosmeceuticals for primary care. The methodology offers a replicable model for scientifically evaluating other classical topical preparations.

### 4.7 Limitations of the study

This exploratory study involved only 30 participants in a single-arm, short-duration design and, therefore, should be regarded as an exploratory study. The participants were all students and staff from the same institution and may not represent the general population, limiting the generalisability of the findings.

The present study has limitations inherent to its exploratory single-arm design. The absence of a control group limits causal interpretation of the observed changes, and the relatively small sample size and short study duration further restrict generalisability. As a pilot proof-of-concept investigation, the findings should be interpreted as preliminary and hypothesis-generating. Future randomized controlled studies with larger sample sizes and longer follow-up periods are needed to validate these observations.

The short duration also restricts understanding of the long-term efficacy and the potential for relapse of pigmentation or erythema after discontinuation. Similarly, parameters such as TEWL and skin thickness, measured over a brief period, may not capture variations that could occur with longer application cycles or under different environmental conditions (e.g., humidity, temperature, or sunlight exposure). Validated patient-reported outcome measures were not documented in this study and are a limitation of this exploratory study.

From an analytical standpoint, our untargeted UPLC–MS/MS QTOF workflow identified only the major known skin-active constituents present in the spectral library. Owing to the inherent limitations of untargeted metabolomics, low-abundance or unidentified compounds might have been missed.

## 5 Conclusion

The findings from this exploratory study underscore the classical wisdom embedded in *Kumkumadi Taila* and its relevance in primary care as a preventive skincare product. The statistically significant improvements in melanin index, erythema, and elasticity highlight the formulation's association with enhancing skin tone, reducing inflammation, and promoting firmness. The absence of adverse effects and the participant-reported tolerability reinforce its safety profile.

Furthermore, the chemical profiling through UPLC-MS/MS QTOF provided crucial insights into the bioactive compounds responsible for its dermatological benefits. The identification of antioxidant, anti-inflammatory, tyrosinase-inhibitory, and photoprotective constituents such as safranal, liquiritin, rubiadin, and nuciferine offers a mechanistic explanation for the observed clinical outcomes and aligns with Ayurvedic descriptions of *Varnya* and *Rakta Prasadana* properties.

This integrative approach, merging classical Ayurvedic therapeutic logic with modern analytical tools and objective skin assessments, validates *Kumkumadi Taila* as a scientifically sound and clinically effective skincare formulation. It provides a template for rigorously assessing, documenting, and positioning traditional topical preparations within evidence-based practice frameworks.

This dual-approach methodology combining objective skin analysis with phytochemical profiling has not been previously reported for any classical Ayurvedic oil formulation and serves as a proof-of-concept.

## 6 Future directions

While the results are encouraging, this exploratory study has clear limitations. The sample size was small and was a pilot study without a control arm. The participants were a relatively homogeneous group from a single institution, which limits the generalisability of the findings. Larger, longer-term trials (≥3 months) would be necessary to assess sustained dermal remodeling and stable melanin modulation. In this study we have selected healthy participants; hence, more diverse populations are needed to confirm the durability and applicability of this approach across various skin types and conditions. Incorporation of validated patient-reported outcome measures in future studies would help to capture subjective perceptions of change among participants. Future work should utilize targeted analytical phytochemical profiling with reference standards to more accurately quantify minor actives (e.g., crocin, santalol, santalin) and compare classical *Kumkumadi Taila* with proprietary variants to establish robust standardization benchmarks. Mechanistic studies on gene expression and signaling pathways, long-term safety and photoprotection evaluations, and testing of modern delivery formats that preserve classical principles while improving usability will be important next steps.

There may be batch-to-batch variations in the quantity of phytochemicals in this formulation, even if procured from the same pharmaceutical company, as the raw materials are likely procured from different sources as per the availability, and this is a common issue faced by the Ayurveda pharmaceutical industry. One potential future approach to address batch-to-batch variability is to conduct bridging clinical studies comparing different production batches to establish consistency in therapeutic response and to define a minimum baseline clinical effect of the formulation.

This study offers a replicable model for evaluating Ayurvedic dermatological therapies with scientific rigor, paving the way for their global mainstream integration.

## Data Availability

The raw data supporting the conclusions of this article will be made available by the authors, without undue reservation when required.
